# Combinatorial biosynthesis of novel aminoglycoside antibiotics via pathway engineering

**DOI:** 10.1186/s13568-024-01753-w

**Published:** 2024-09-16

**Authors:** Khaled M. Aboshanab, Mohammad Y. Alshahrani, Ahmed Alafeefy

**Affiliations:** 1 Department of Microbiology and Immunology, Faculty of Pharmacy, Organization of African Unity St, Ain Shams University, 11566 Cairo, Egypt; 2 Department of Clinical Laboratory Sciences, College of Applied Medical Sciences, King Khalid University, P.O. Box 61413, 9088 Abha, Saudi Arabia; 3 Faculty of Pharmacy, University Technology MARA (UiTM), Campus Puncak Alam, Bandar Puncak Alam, Puncak Alam, 42300 Selangor Malaysia

**Keywords:** Pathway engineering, Combinatorial biosynthesis, Aminoglycoside, Gene clusters, Mutations

## Abstract

With the current spread of clinically relevant multidrug-resistant (MDR) pathogens, insufficient unearthing of new anti-infectives, and the high cost required for approval of new antimicrobial agents, a strong need for getting these agents via more economic and other alternative routes has emerged. With the discovery of the biosynthetic pathways of various antibiotics pointing out the role of each gene/protein in their antibiotic-producing strains, it became apparent that the biosynthetic gene clusters can be manipulated to produce modified antibiotics. This new approach is known as the combinatorial biosynthesis of new antibiotics which can be employed for obtaining novel derivatives of these valuable antibiotics using genetically modified antibiotic-producing strains (pathway engineering). In this review and based on the available biosynthetic gene clusters of the major aminoglycoside antibiotics (AGAs), the possible alterations or modifications that could be done by co-expression of certain gene(s) previously known to be involved in unique biosynthetic steps have been discussed. In this review defined novel examples of modified AGA using this approach were described and the information provided will act as a platform of researchers to get and develop new antibiotics by the antibiotic-producing bacterial strains such as *Streptomyces*, *Micromonospora*,…etc. This way, novel antibiotics with new biological activities could be isolated and used in the treatment of infectious diseases conferring resistance to existing antibiotics.

**Supplementary Information:**

The online version contains supplementary material available at 10.1186/s13568-024-01753-w.

## Introduction

With the current development of microbial resistance toward most of the antibiotics in practice and the spread of clinically relevant MDR pathogens, the inadequate finding of new antibiotics, and the great cost required for preclinical and clinical evaluation of newly discovered antibiotics, a strong need for getting these agents via more economic and other alternative new route has emerged. Recently, with the extensive knowledge and profound use of gene manipulations, it became apparent that this new field could be used and designed in such a way that helps in solving the current problem of finding modified members of clinically used antibiotics (Terreni et al. 2021; Santos-Beneit 2024). This new technique is currently known as the combinatorial formation of new antibiotics or pathway engineering. It can be considered as one of the most important tools for getting newly modified compounds using genetically modified antibiotic-producing strains. This approach includes directing the antibiotic-producing cells toward the production of altered or modified antibiotics that have never existed (Atanasov et al. 2021; Cook and Stasulli 2024). This approach can be achieved through alteration of the biosynthetic gene clusters that are involved in the biosynthesis of such antibiotics via site-directed mutagenesis, protoplast fusion, gene replacement, inversion, inactivation through knock-out mutations, or co-expression of certain gene(s) previously known to be involved in unique biosynthetic steps (Liu et al. 2017; Miethke et al. 2021; Kamel et al. 2022; Mahdizade Ari et al. 2024). Such modifications are mostly rationale-based i.e. deletion of the genes responsible for the synthesis of a certain chemical moiety that can be used as a target for bacterial resistance. This way, new antibiotics with new biological activities could be obtained and used in the treatment of infectious diseases conferring resistance to the existing antibiotics (León-Buitimea et al. 2022; Mahdizade Ari et al. 2024).

**Fig. 1:**
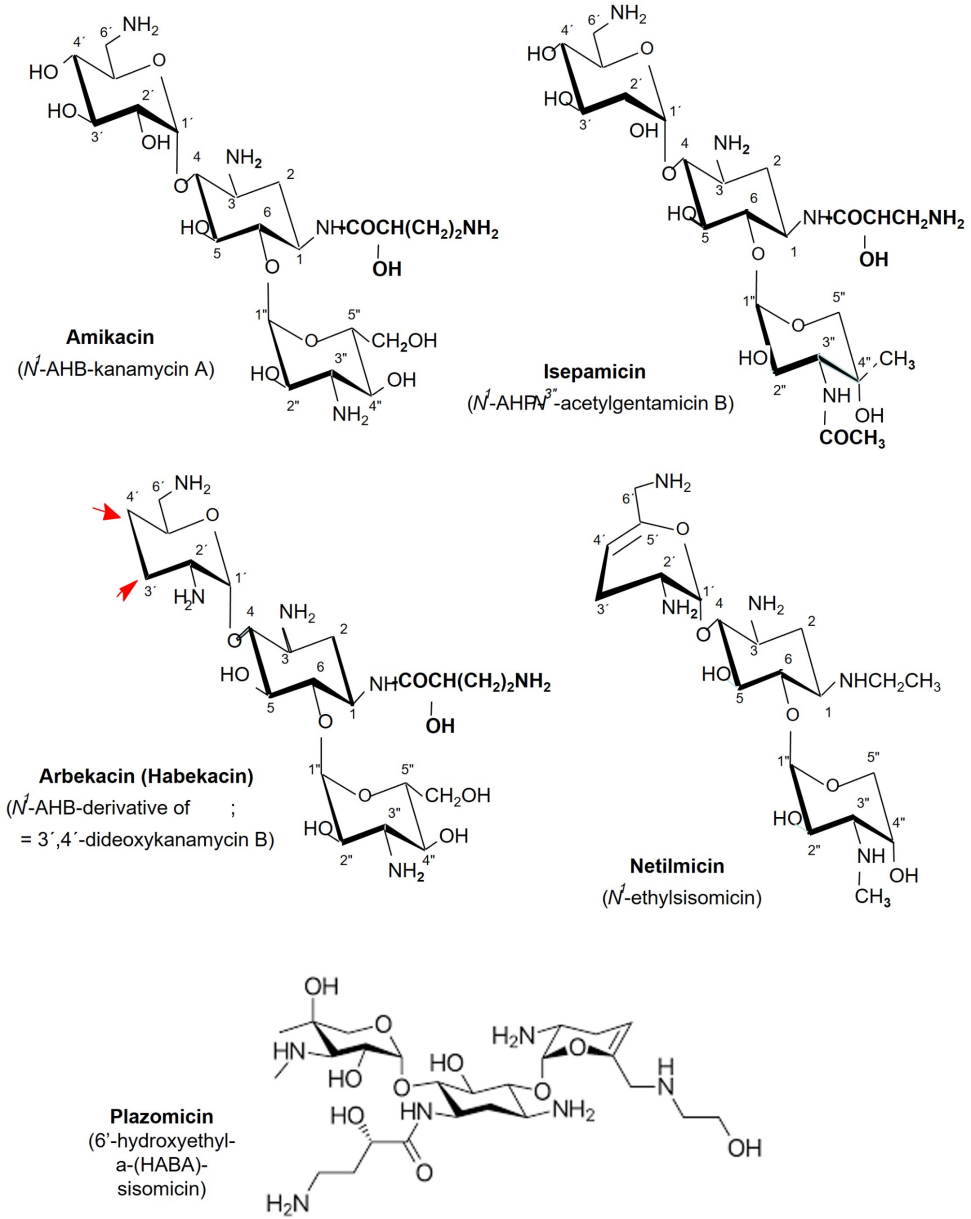
Chemical structures of semi synthetic aminocyclitol aminoglycoside antibiotics (ACAGAs)

Most of the aminoglycoside antibiotics are products of higher actinomycetes. Among these the kanamycins (KM) and gentamicins (GM) are clinically active aminoglycoside of the 2-deoxystreptamine (2DOS; 1,2,3-trideoxy-1,3-diamino-scyllo-inositol) containing aminocyclitol-aminoglycoside antibiotics (ACAGAs) (Kirst and Marinelli 2014; Lyu et al. 2024; Oh et al. 2007). KMs are produced by *Streptomyces* (*S*.) sp., GMs by various strains of *Micromonospora* sp. These two families of compounds share the structural feature of being 4,6-disubstituted (by glycosyl residues) 2DOS derivatives and are pseudo-trisaccharides. The biosynthetic pathways of various ACAGAs where the intermediates, 2-deoxy streptamine and paromamine are indicated is delineated in Fig. S1. They are composed of many individual variants which either are end or side products of the natural biosynthetic pathways (e.g. KM A, B; tobramycin; nebramycin 4, 5; GM C-complex; sisomicin; verdamicin; sagamicin. Furthermore, these antibiotics have been semi-synthetically modified (e.g. amikacin, habekacin, isepamicin or netilmicin) to yield derivatives with better pharmacological properties or/and being inert to wide-spread resistance mechanisms (Lyu et al. 2024) (Fig. 1).

The two-aminocyclitol aminoglycoside antibiotic (ACAGA) families, especially the GMs, share some of their features with another aminoglycoside class, the fortimicins (FMs; produced by *Micromonospora* sp.) and istamycins (IMs; produced by *Streptomyces* sp.), which are pseudo disaccharides and contain a different type of diaminocyclitol, the monosubstituted derivatives of 3,6-dideoxy-3,6-diamino-*neo*-cyclitol. All three groups have a very closely related mode of action and share a target site (16 S ribosomal RNA) on the small subunits of bacterial ribosomes; this is reflected also in the common resistance mechanism; a KM/GM/FM-resistance determining 16 S rRNA methyltransferase, by which the actinomycete producers protect themselves (Oh et al. 2007). Members or derivatives of these three groups of ACAGAs have been used in several fields of applications, mainly in the control and management of severe nosocomial illnesses produced by Gram-negative opportunistic bacteria in immuno-compromised patients.

Before applying pathway engineering, a full in-depth knowledge about the genetics, biosynthesis, regulation, and transport of these metabolites that occur inside the producing cell is necessary. Among the several classes of antibiotics that have already been known, the proposal of the current project is mainly directed towards a very important class of broad-spectrum bactericidal antibiotics, namely aminoglycoside antibiotics. The reasons attributed to the selection of this class could be summarized as follows: (i) most of the biosynthetic gene clusters of the major members of these antibiotics have been partially or fully sequenced and analyzed by several investigators (Kharel et al. 2004a, b; Subba et al. 2005; Kirst and Marinelli 2014; Piepersberg et al. 2007; Wehmeier and Piepersberg 2009) (Table 1); (ii) a reasonable amount of biochemical information on their production and regulation are available (Piepersberg et al. 2007); (iii) enough information on their resistance in either their producers or clinically relevant pathogens are almost known (Behera et al. 2023; Hamed et al. 2018) (iv) the molecular characteristics of interaction with cellular entities are well studied (Liu et al. 2022); (v) novel era of application and new biologically relevant targets have emerged (Khan et al. 2020).

**Table 1:** The biosynthetic gene clusters and NCBI accession codes of the major aminocyclitol aminoglycoside antibiotics deposited in the NCBI GenBank database

ACAGAs (abbreviation of the gene cluster)	ACAGA-producing strain number	NCBI GenBank accession code(s)
kanamycin (*kan*)	*S. kanamyceticus* DSM 40,500	AJ628422, AJ582817.3, AB254082, AB254080, AB164642.1, AJ628422.2
neomycin (*neo*)	*S. fradiae* DSM 40,063	AJ629247, KX871906, AJ843080, AJ786317
paromomycin (par)	*S. rimosus* subspecies *paromomycinus* NRRL 2455	AJ628955
tobramycin (*tob*)/apramycin (*apr*)	*S. tenebrarius* DSM 40,477	AJ810851, AJ579650, AJ629123, AJ575934
hygromycin (*hyg*)	*S. hygroscopicus* subspecies *hygroscopicus* DSM 40,578	AJ628642., DQ314862
ribostmaycin (*rib*)	*S. ribosidificus* NRRL B-11,466	AJ744850, AJ748131
lividomycin (*liv*)	*S. lividus* ATCC 21,178	AJ748832
istamycin (*ist*)	*S. tenjimariensis* ATCC 31,603	AJ845083
tobramycin (*apr*)	*Streptoalloteichus hindustanus* DSM 44,523	AJ875019, AB103327
gentamicin (*gen*)	*M. echinospora* DSM 43,036	AJ628149, JQ975418, AJ575934.3
fortimicin (*for*)	*M. olivasterospora* DSM 43,868	AJ628421
butirosin (*btr*)	*B. circulans* ATCC 21,558	AJ781030, LC571042, AJ847918, AB097196

*apr* = apramycin; *btr* = butirosin; *for* = fortimicin; *gen* = gentamicin; *hyg* = hygromycin B; *ist* = istamycin; *kan* = kanamycin; *liv* = lividomycin; *M. = Micromonospora;* NCBI = National Centre for Biotechnology Information; *neo* = neomycin; *par* = paromomycin; *rib* = ribostamycin; *S.* = *Streptomyces; tob* = tobramycin; ATCC = American Type Culture Collection; DSM = Deutsche Sammlung von Mikroorganismen und Zellkulturen GmbH. NRRL = Northern Regional Research Laboratory

ACAGAs are known to be the leading representatives of the aminoglycoside group, and they comprise several subclasses that are classified based on the chemical nature of the aminocyclitol moiety, the basic aglycone unit in all the ACAGAs. They comprise (i) tryptamine-containing ACAGAs such as streptomycin, bluensomycin, ashimycin A and B, and spectinomycin (Piepersberg et al. 2007); (ii) 2-deoxystreptamine-containing ACAGAs (2DOS-ACAGAs) which includes a relatively homogeneous biosynthetic group such as the neomycins (NMs), kanamycins (KMs) and gentamicins (GMs) as well as the more distantly related antibiotics, apramycin (Apr) and hygromycin B (HM-B) (Piepersberg et al. 2007; Wehmeier and Piepersberg 2009), (iii) fortamine and 2-deoxyfortamine-containing ACAGAs including fortimicins (FMs) and istamycin (IM), containing fortamine and 2-deoxyfortamine as a prime cyclitol entity, respectively.

In this review, we deduced a strategy that will allow researchers to find new antibiotics with new biological activities and gather in-depth knowledge about their biosynthesis, particularly on both the biochemical and genetic levels. Moreover, in this review, researchers will gain information to be used as a platform for future studies. These future aspects include: (i) combinatorial biosynthesis of new members of ACAGAs with new biological activities; (ii) profound handling and use of genetic material in the way that it attributes to overcome the widespread microbial resistance; (iii) the successful genetic manipulation of the gene products that are involved in the biosynthesis of ACAGAs; and (iv) statistically optimizing production of the target antibiotics by their natural producers through physiological optimization technique, also, known as response surface methodology (RSM) as well as using different genetic methods such as gene duplication, homologous and heterologous expression of certain key genes (Ibrahim et al. 2019, 2023; El-Housseiny et al. 2021). This, of course, would be designed in such a way not only to increase the yield but also to improve the microbial profile of the existing antibiotics.

Furthermore, the key naturally derived medications have been developed by managing the formation of secondary metabolites for combating clinically relevant pathogens such as mycobacterial infections (Zhu et al. 2019)or their virulence such as biofilm formation (Zhou et al. 2018; Khan et al. 2020). Recent developments in combinatorial biosynthetic techniques have improved natural product structural diversity to a great extent. Actinomycetes produce a significant family of antibiotics known as aminoglycosides (AGs). However, because of their unwanted toxicity and frequent resistance, AGs have had a significant negative impact on therapeutic uses. The primary reason these incredibly strong AGs are hazardous is because they accidentally attach to the vital eukaryotic ribosome. Recently, the multicomponent toxicity of kanamycin A was effectively investigated using a bioassay-guided plasma metabolomics technique, which may aid in further investigating the side effects (Atanasov et al. 2021).

A viable approach to overcoming these difficulties is the investigation of AGAs structural analogues with new biological activity and enhanced pharmacological characteristics. The growing use of combinatorial approaches presents extraordinary chances to find untapped natural chemicals, particularly difficult ones like aminoglycoside antibiotics. Using naturally occurring actinomycetes that produce AGAs, conventional genetic procedures are employed to change the gene clusters responsible for AGAs production (Atanasov et al. 2021; Ban et al. 2021). However, the precursor supply of AGAs can be completely stopped in the *Streptomyces* cell factory without resulting in any resistance indicators by using the CRISPR/Cas9-mediated chromosomal editing technique. It is impossible to overstate the potential of combinatorial biosynthesis in the search for aminoglycoside antibiotics (Tao et al. 2018). Accordingly, this review concentrates on the important and therapeutically significant aminoglycoside antibiotics that are currently utilized in clinical practice: the 2-DOS containing ACAGAs. In order to produce more potent antibiotics that either do not contain the target groups or have these targets protected from the action of aminoglycoside-modifying enzymes produced by bacterial pathogens, this review focusses on specific modifications in the biosynthetic routes of the respective antibiotics in their natural producers(Wang et al. 2022; Thy et al. 2023). For instance, the antibiotic’s effectiveness against bacteria that make phosphotransferase will remain intact if the 3’-OH group, which is the target group for phosphotransferase of adenyl transferase produced by clinically relevant pathogens, is removed. Furthermore, the hydroxybutyric moiety’s transfer to safeguard the amino group at position 1 of the aglycone moiety will prevent the antibiotic from being acylated by bacterial pathogen-produced acetyltransferase (d’Udekem d’Acoz et al. 2024; Sklenicka et al. 2024). Also, in this review we described defined examples of such modifications and such information will help researchers to get and develop new antibiotics by the antibiotic-producing bacterial strains such as *Streptomyces*, *Micromonospora*,…etc.

## Overview of aminoglycoside antibiotics

Using a combinatorial biosynthesis approach, aminoglycosides are produced from primary metabolites including L-glutamine, D-glucosamine, and L-2-deoxystreptamine (2-DOS). They consist of one or two amino sugars at D-glucosamine’s C-4 and C-6 and two sugar moieties, namely D-glucosamine (Mikolasch et al. 2022). Different glycosyltransferases convert these sugar moieties from uridine diphosphate (UDP)-sugar derivatives to their common intermediates (neomycin, paromomycin, or kanamycin). The biosynthesis process is complicated, and producing novel aminoglycosides by biosynthetic engineering is a difficult undertaking because of this. There are also many enzymes involved in this system (Yu et al. 2017). Utilizing promiscuous or engineered enzymes that can use a broad range of substrates is one way to control the biosynthetic pathway for aminoglycosides of interest, as the vast majority of the enzymes involved in aminoglycoside biosynthesis are fairly specific for their natural substrates (Yu et al. 2017). In particular, only modification enzymes are considered for engineering techniques, such as adenylyltransferase (ANT), phosphotransferase (APH), acetyltransferase (AAC), and glycosyltransferase (GT) (Thacharodi and Lamont 2022).

To tackle the recently increasing antibiotic resistance, these antibiotics could be further modified in addition to creating the modification enzymes for aminoglycosides. Finding effective NDP-sugar donors is the hardest aspect of the combinatorial biosynthesis of aminoglycosides. Generally, two cooperative enzymes—an NDP-generating enzyme and a sugar-1 phosphoryltransferase—are used to biosynthetically engineer the NDP-sugars. This calls for many steps and a wide degree of specificity (Thacharodi and Lamont 2022). In addition, the obstacles that must be overcome for biosynthetic engineering in the areas of combinatorial efficiency management, NDP-sugar synthases, and NDP-sugar-modifying enzymes has been discussed in this mini-review. With the recent development of effective NDP-sugar synthases by mutagenesis and broad promiscuous enzyme identification, glycosylation pathway engineering can now be used to obtain novel antibiotics throughout new phases of drug screening (Thacharodi and Lamont 2022).

## The gene clusters of the neomycin family

The NMs are a sub-class of the 2-deoxystreptamine (2DOS; 1,2,3-trideoxy-1,3-diamino-*scyllo*-inositol) containing aminocyclitol-aminoglycoside antibiotics (ACAGAs) with an exceptional range of spread and variance as compared to other related ACAGAs (Piepersberg et al. 2007; Seghezzi et al. 2013; Subramani et al. 2023; Veirup et al. 2024; Wehmeier and Piepersberg 2009). They are not only produced in actinomycetes (genera *Streptomyces* and *Micromonospora*, but also in bacilli (*Bacillus circulans*). Their structural variants form product families which have been called neomycins (NMs; producers are *S*. *fradiae* and *M*. *chalcea*), ribostamycin (RMs; producer is *S*. *ribosidificus*), paromomycins (PMs; *S*. *rimosus* ssp. *paromomycinus*), lividomycins (LMs; *S*. *lividus*), and butirosins (BUs; *B*. *circulans*) (Kharel et al. 2004a, b; Subba et al. 2005). Beyond being formed via a paromamine intermediate (D-glucosamine-alpha-1,4-2DOS), as are the KMs and GMs, these natural compounds are characterized by their second-step glycosylation at the C5-hydroxyl of the 2DOS moiety with a ribosyl residue (D-glucosamine-alpha-1,4-2DOS-beta-5,1-D-ribose). This core pseudo trisaccharide becomes further modified by another glycosylation step (D-glucosamine-alpha-1,4-2DOS-beta-5,1-D-ribose-alpha-3,1-D-glucosamine; in the NMs, PMs, and LMs) and/or a series of tailoring reactions characteristic for the individual family; these latter are (i) 6’- and 6’’’-amination and 5’’’-epimerization (in the NMs), (ii) 6’-amination (in RM), (iii) 6’’’-amination and 5’’’-epimerization without 6’-amination (in the PMs), (iv) 3’-dehydroxylation, 6’’’-amination and 5’’’-epimerization without 6’-amination (in the LMs), (v) 6’-amination, 3’’-epimerization, and 1-*N*-acylation with AHBA ([2R]-4-amino-2-hydroxybutyric acid; in the BUs) (Kharel et al. 2004a; Subba et al. 2005; Wehmeier and Piepersberg 2009). All the members of the neomycin subclass of ACAGAs have lethal effects on their target bacteria, act on the bacterial translation apparatus, induce misreading of the genetic code, and bind specifically to a particular structure in the 16 S rRNA of the small (30 S) subunit of the bacterial ribosome (Veirup et al. 2024).

NMs and PMs have found a wider use in clinical applications and BUs gave a paradigm to produce successful semisynthetic ACAGAs like amikacin and arbekacin (Inoue et al. 1994). However, side effects and increasing occurrence and dissemination of various resistance determinants have caused ceasing use of these compounds. The most dominant resistance genes encode modifying enzymes of the types of aminoglycoside phosphotransferase (APH-3’) and aminoglycoside acyltransferase (AAC-3), encoded by *aphA* and *aacC* genes, respectively, and are found as resistance mechanisms in the producers themselves (Salauze et al. 1991). There are, however, indications that these basic resistance determinants have been left with further metabolic/structural re-design of some of the NM-related compounds of this family: especially the LMs, lacking a 3’-hydroxyl, cannot be inactivated by the APH (3’)-type of modifying enzymes anymore. Also, there were already incidences for the *aacC* genes from NM-producing *S*. *fradiae* and from PM-producing *S*. *rimosus* sp. *paromomycinus* did not occur in a conserved genetic environment, i.e. either of these genes seemed to be located outside the producing gene cluster for the compound itself (Salauze et al. 1991; Wehmeier and Piepersberg 2009).

The gene order and positions of the *neo*-, *rib*-, *par*-, *liv and btr* clusters (that are involved in the biosynthesis of NMs, RMs, PMs, LVs and BUs, respectively) investigated can be abridged as follows: (i) the *neo*-, *rib*-, *par*-, and *liv*-clusters are remarkably homogenous in DNA composition (G + C content between 72.1 (*rib)* and between 76.8 (*neo*), sequence similarity (Wehmeier and Piepersberg 2009) and gene order (Piepersberg et al. 2007); (ii) the *btr*-cluster from *B*. *circulans* diverges from this pattern, though most or all of the genes necessary for the basic 2DOS and ribostamycin pathways seem to be conserved (Piepersberg et al. 2007); (iii) a bigger divergence exists in both the content and order of genes and the composition and sequence similarity of the respective DNA segments between the streptomycete and bacilli (*B*. *circulans*) sources of AGA clusters(Piepersberg et al. 2007).

## The biosynthetic pathways of neomycins (NMs), ribostamycin (RM), promomycins (PMs), lividomycins (LMs), and butirosins (BUs)

Obviously, the genes involved in the biosynthetic pathways for the neomycin-related 2DOS-ACAGAs appear to correspond to a more basic metabolic invention yielding this class of natural products and could have evolved a much longer time ago, than e.g. those for the KMs, GMs, and FTs (Wehmeier and Piepersberg 2009). The factors and evidence attributed to this observation originate from the following proofs: (i) the gene clusters of this family are present in taxonomically distant bacterial species including, *Bacillaceae*, *Streptomycetaceae*, and *Micromonosporaceae*; (ii) a lower number of the genes that are involved in the biosynthesis of basic aglycone moiety (2DOS/paromamine) as compared to the KM- and GM-pathways; (iii) well-maintained and comparable genes show a higher degree of divergence was observed in homologous genes (genes putatively involved in the same biosynthetic step); (iv) absence of gene duplications as compared to other gene clusters such as KM- or GM-gene clusters; (v) all encoding aminoglycoside 3’-phosphotransferases as resistance genes (*aphA* genes), instead of having additional genes that are involved in dehydroxylation processes.

## The basic pathways for making the 2-deoxy streptamine (2DOS) aminocyclitol and the paromamine precursor

The biochemistry and function of 2-deoxy-*scyllo*-inosose synthases or cyclases (NeoC and its homologs), L-glutamine: ketocyclitol aminotransferases I and II (NeoS and its homologs) and cyclitol [1-]dehydrogenases (neoE and its homologs), have previously been confirmed by several researchers (Ahlert et al. 1997; Kudo et al. 1999; Kudo and Eguchi 2009; Nango et al. 2008). Whether the ForS aminotransferase is also a bifunctional enzyme and responsible for the introduction of the second amino group in the 6-position (according to the cyclitol nomenclature), as in the 2DOS seems likely, is still questionable. This is because a second candidate gene is lacking; however, this will have to be elucidated by mutant and biochemical studies. The pathway in the respective ACAGA-producers begins with a glycosyl transfer reaction catalyzed by glycosyltransferases (NeoM and its homologous) of the D-glucosamine unit to the aglycone (2DOS or *scyllo*-inosamine), forming a pseudodisaccharide intermediate, paromamine (in the NM, KM and GM pathways) and fortimicin FU-10 in that for the FMs, respectively (Wehmeier and Piepersberg 2009).

Cluster-specific regulation of the genes in the actinomycete clusters for NM-type AGAs is likely and could depend on a new type of secondary metabolic sensor-response regulator system composed of three instead of the usually two protein components: the G/H/I-sets of conserved proteins encoded in the *neo*-, *rib*-, par-, and *liv*-clusters. Genes for these three highly conserved protein families also are found in the KM- (*kan* gene cluster), hygromycin B- (*hyg*), and cinnamycin-clusters (Widdick et al. 2003). The NeoI/H/G-related protein sets could represent a conserved set of protein components forming new putative regulatory complexes like the sensor kinase/response regulator systems (Galperin 2004). Proof for this speculation comes from the following: (1) In the *neo*-, *rib*-, *par*-, and *liv*-clusters, but also in those for the aminoglycosides kanamycin (*kan*) and hygromycin B (*hyg*) (Piepersberg et al. 2007), as well as in the cinnamycin gene cluster of *S*. *cinnamoneus* ssp. *cinnamoneus* DSM 40,005 (Wehmeier and Piepersberg 2009; Widdick et al. 2003). Three intensely preserved reading frames, which obviously mostly arise in a common operon (exception: *parI* lies separate from *parHG*). Corresponding genes are lacking from other related aminoglycoside clusters in actinomycetes, such as those to produce GM, FM, tobramycin (TM) and apramycin (AM) (Piepersberg et al. 2007; Wehmeier and Piepersberg 2009).

### Enzymes involved in the biosynthesis of the major aminocyclitol aminoglycoside antibiotics (ACAGAs)

Several studies have been conducted to explore the biochemistry of the various gene products/enzymes in the biosynthesis of the major ACAGs including descriptions of some fundamental enzymes in various pathways (Wehmeier and Piepersberg 2009; Yu et al. 2017). Therefore, protein pathway engineering of ACAGAs can be considered a promising strategy to get newly discovered antibiotics to combat the clinically relevant MDR pathogens and overcome the newly emerged pathogens (Aggen et al. 2010; Armstrong and Miller 2010; Sader et al. 2023). Interestingly, plazomicin is a new semisynthetic ACAGA designed by modifying sisomicin with the addition of a 2(S)-hydroxy aminobutyryl group at the N1 position and a hydroxyethyl substituent at the 6′ position (Aggen et al. 2010; Armstrong and Miller 2010) to control and eradicate most of the clinically relevant pathogens and was recently clinically approved (Jung and Gademann 2023; Sader et al. 2023). Plazomicin showed a marked activity more than amikacin, GM, or TM against MDR Enterobacterales (Sader et al. 2023) and was shown to retain its activity against CRE (94.0%S), extended-spectrum beta-lactamase (ESBL)-producing (98.9%S), and MDR (94.8%S) clinical isolates (Sader et al. 2023).

### Examples of pathway engineering to obtain novel aminoglycoside antibiotics (AGAs)

Based on the described biosynthetic pathways and on the enzymes that have been proven to catalyze certain biosynthetic steps of the major ACAGS, we proposed combinatorial biosynthesis of some novel ACAGAs as follows:

### Combinatorial biosynthesis of amikacin from butirosin B (BT-B)

**Fig. 2:**
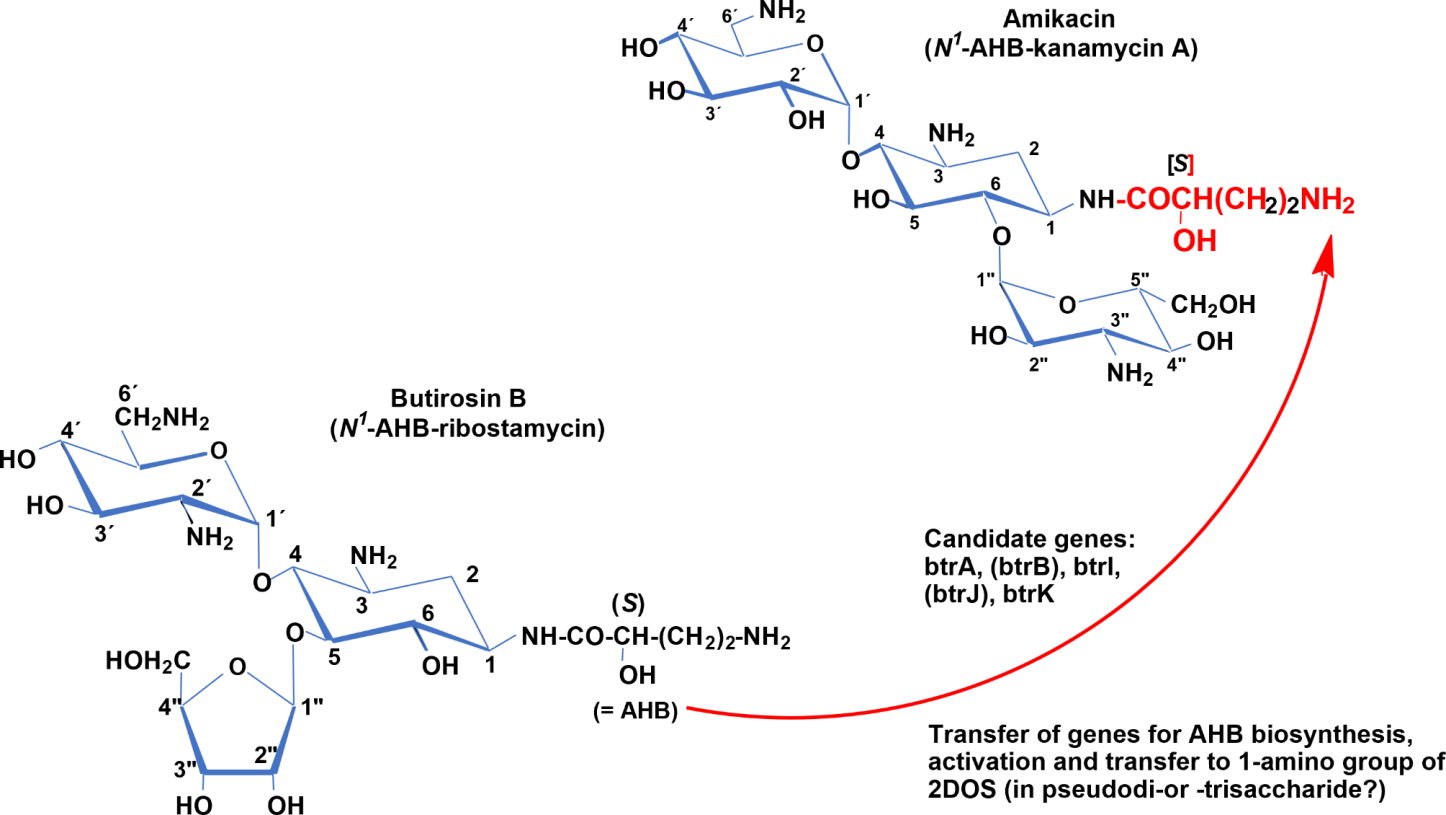
Combinatorial biosynthesis of amikacin from butirosin B (BT-B). BtrA. BtrB, BtrI, BtrJ, BtrK are enzymes derived from the biosynthetic gene cluster of butirosin (NCBI accession code, AB097196) are involved in the transfer of the aminobutyryl moiety to the amino group located at position 1 of aglycone moiety

Amikacin is a semisynthetic ACAGAs which is produced from KM-A after certain chemical modifications (Kawaguchi et al. 1972). However, it can be produced naturally through a heterologous expression of the genes/proteins (BtrA, BtrB, BtrI, BtrJ and BtrK) into *S. kanamyceticus* DSM 40,500, a producer of KM-A (Fig. 2). The respective genes/proteins are involved in the biosynthesis of amino hydroxybutyrate (AHB) moiety in *Bacillus circulans* ATCC 21,558 as previously reported (Aubert-Pivert et al. 1993; Llewellyn et al. 2007).

## Combinatorial biosynthesis of N1-AHB-Neomycin B (N1-AHB-NM-B)

**Fig. 3:**
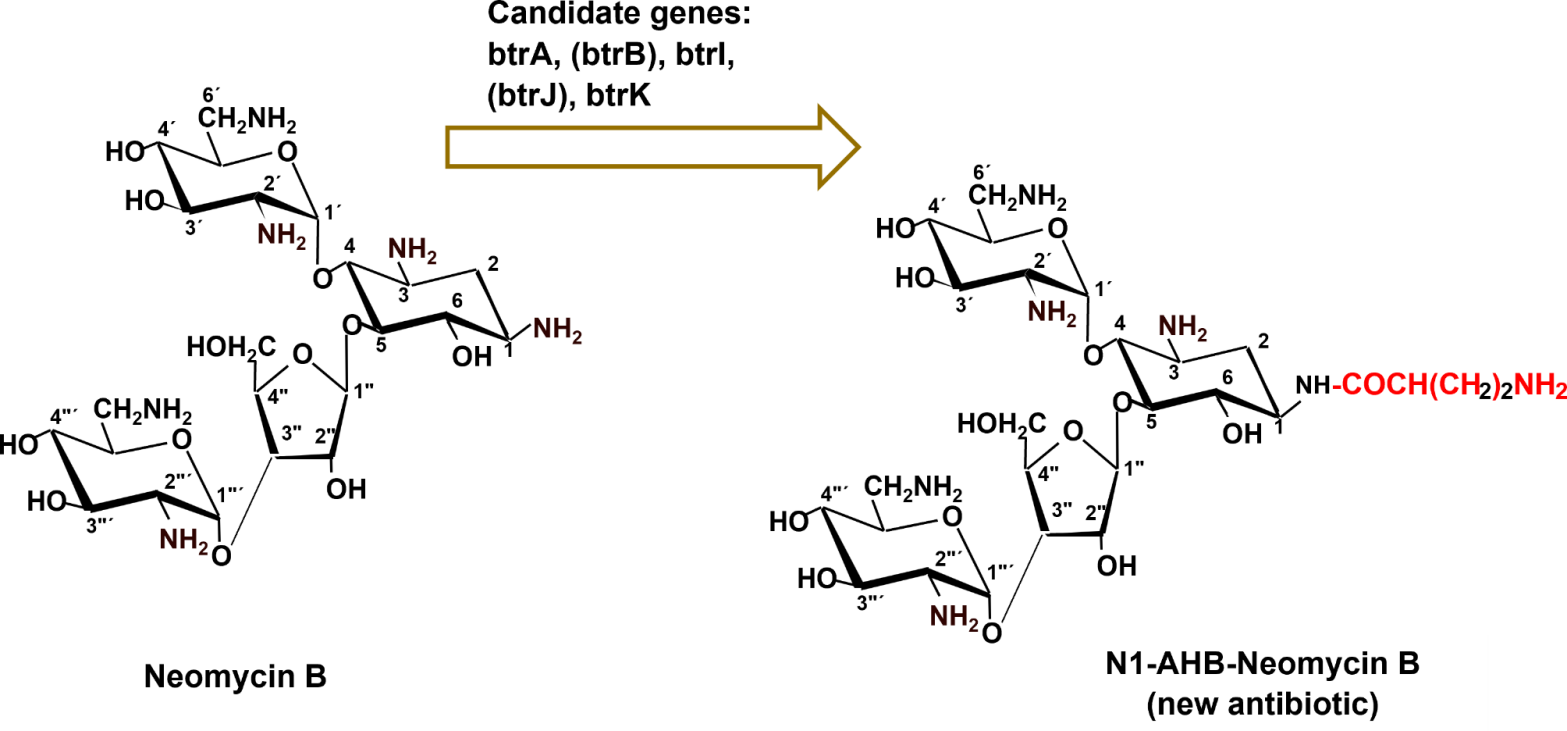
Combinatorial biosynthesis of N1-AHB-Neomycin B (N1-AHB-NM-B). BtrA. BtrB, BtrI, BtrJ, BtrK are enzymes derived from the biosynthetic gene cluster of butirosin (NCBI accession code, AB097196) are involved in the transfer of the aminobutyryl moiety to the amino group located at position 1 of aglycone moiety

As illustrated in Fig. 3, the formation of N1-AHB-Neomycin B can be done by heterologous expression of the genes/protein (BtrA, BtrB, BtrI, BtrJ and BtrK) involved in the biosynthesis of amino hydroxybutyrate (AHB) moiety in *B. circulans* ATCC 21,558 into *S. fradiae* DSM 40,063, a producer of neomycin B. The newly N1-AHB-Neomycin B antibiotic is expected to be more active as compared to neomycin B against MDR pathogens as the amino group at position 1 of the 2DOS will be protected from being acetylated or inactivated by the acetyltransferase-producing bacterial pathogens.

## Combinatorial biosynthesis of 3’-deoxy-neomycin B (3’-deoxy NM-B)

**Fig. 4:**
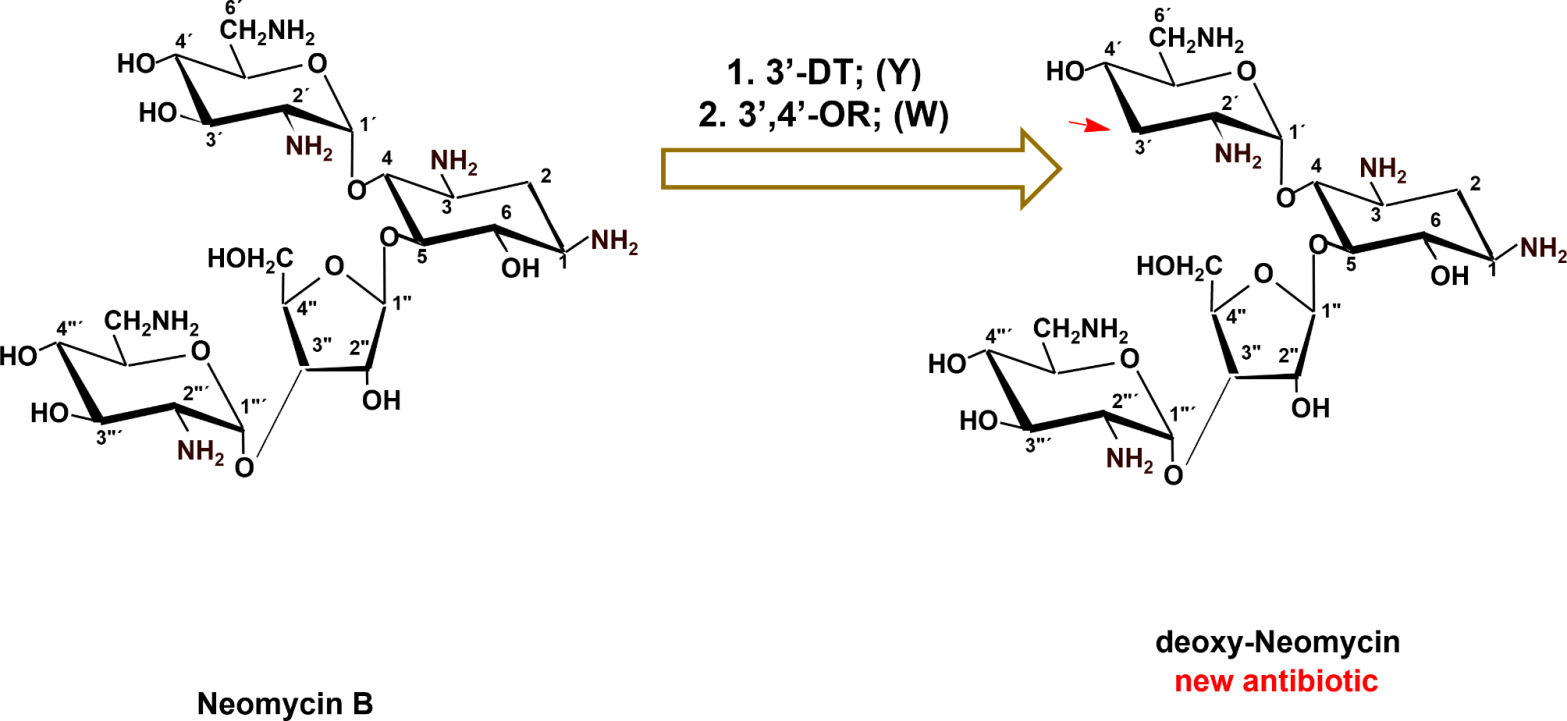
Combinatorial biosynthesis of 3’-deoxy-neomycin B (3’-deoxy NM-B). 3’-DT = 3’ dehydratase (LivY); 3’,4’-OR, 3’,4’-oxidoreductase (LivW) of the lividomycin biosynthetic gene cluster (NCBI accession code, AJ748832

As displayed in Fig. 4, the formation of 3’-deoxy-neomycin B can be done by heterologous expression of the genes/protein (3’-dehydratase (LivY) and 3’-4’ oxidoreductase (LivW) involved in the 3’ deoxygenation processes in *S. lividus* ATCC 21178 (a producer of lividomycin) into *S. fradiae* DSM 40063 (a producer of neomycin B). The newly 3’-deoxy-neomycin B antibiotic would expect to be more active as compared to neomycin B against MDR pathogens as it lacks the 3’-hydroxyl moiety, and this will be protected from being phosphorylated or inactivated by the phosphotransferase transferase-producing bacterial pathogens.

### Biosynthesis of lividomycin (LM) by new route (modified pathway of ***S. rimosus ***NRRL 2455, a producer of paromomycin)

**Fig. 5:**
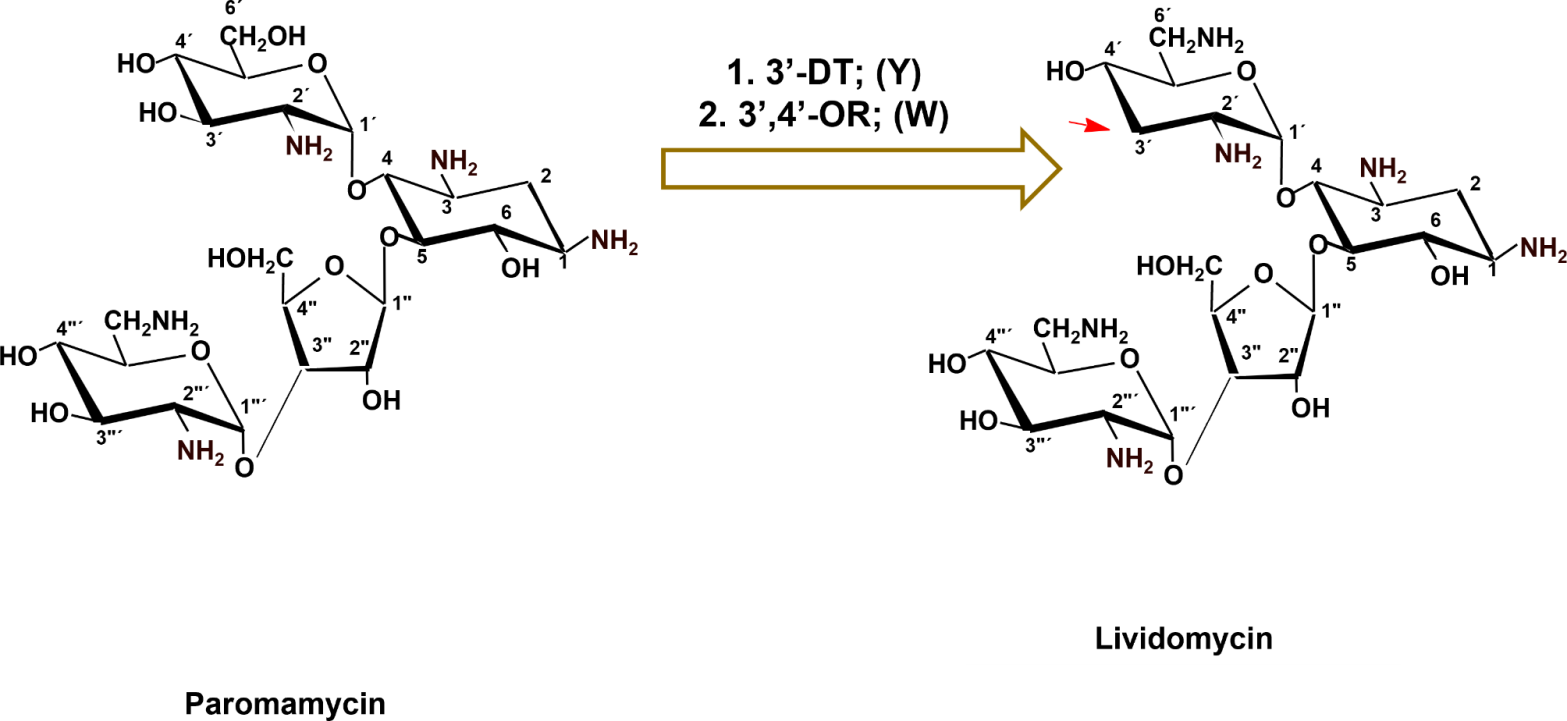
Combinatorial biosynthesis of lividomycin (LM) by new route (modified pathway of *S. rimosus* NRRL 2455, a producer of paromomycin). 3’-DT = 3’ dehydratase (LivY); 3’,4’-OR, 3’,4’-oxidoreductase (LivW) of the lividomycin biosynthetic gene cluster (NCBI accession code, AJ748832

Based on the chemical structure, LM is a 3’-deoxy-paromomycin. Therefore, if the genes/proteins involved in the 3’-dehydroxylation processes such as 3’-dehydratase (LivY) and 3’-4’ oxidoreductase (LivW) in *S. lividus* ATCC 21,178 (Wehmeier and Piepersberg 2009) are heterologously expressed in *S. rimosus* subsp. *paromomycinus* NRRL 2455, a producer of paromomycin (PM), will result in the biosynthesis of LM by *S. rimosus* subsp. *paromomycinus* NRRL 2455 (Fig. 5).

### Combinatorial biosynthesis of 3’-deoxy-ribostamycin (3’-deoxy RM)

**Fig. 6:**
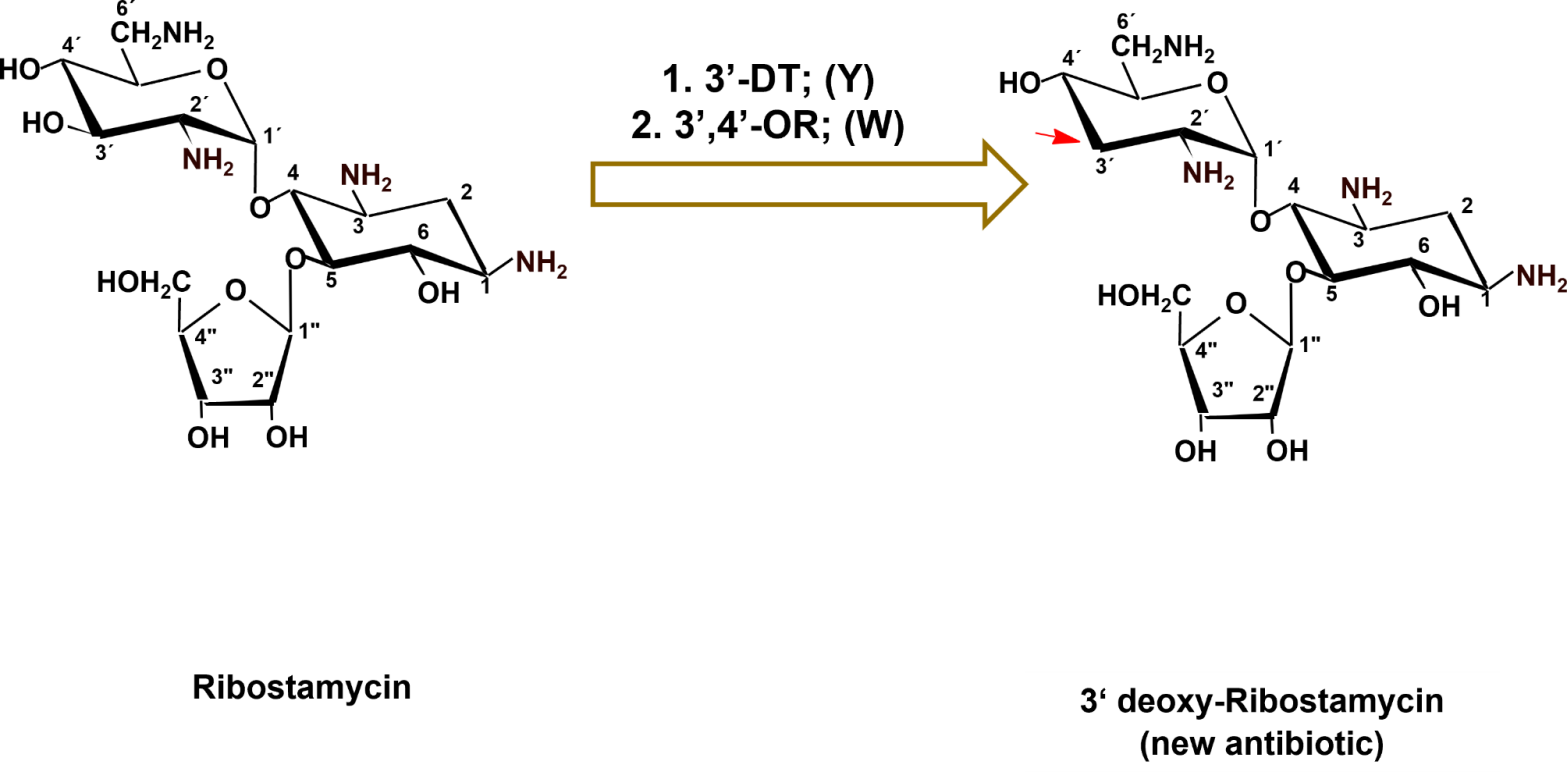
Combinatorial biosynthesis of 3’-deoxy-ribostamycin (3’-deoxy RM). 3’-DT = 3’ dehydratase (LivY); 3’,4’-OR, 3’,4’-oxidoreductase (LivW) of the lividomycin biosynthetic gene cluster (NCBI accession code, AJ748832

As depicted in Fig. 6, the formation of 3’-deoxy-RM can be done by heterologous expression of the genes/protein (3’-dehydratase (LivY) and 3’-4’ oxidoreductase (LivW) involved in the 3’ deoxygenation processes in *S. lividus* ATCC 21178, a producer of LM into *S. ribosidificus* NRRL B-11466, a producer of RM. The newly 3’-deoxy-RM antibiotic is expected to be more active as compared to RM against MDR pathogens as it lacks the 3’ hydroxyl moiety and this will be protected from being phosphorylated or inactivated by the phosphotransferase transferase-producing pathogens. In conclusion, **t**his study highlights the combinatorial biosynthesis of ACAGAs via pathway engineering which can be accomplished through heterologous expression of certain genes/proteins previously verified to be involved in certain catalytic steps in the biosynthetic pathways of the respective antibiotics. This approach can be considered one of the most important tools for getting new valuable antibiotics using genetically modified antibiotic-producing strains. This way, numerous novel antibiotics with new biological activities could be isolated and used in the treatment of infectious diseases conferring resistance to existing antibiotics.

## Electronic supplementary material

Supplementary Material 1

## Data Availability

All data generated or analyzed during this study are included in this published article. The analyzed biosynthetic gene clusters of the aminocyclitol aminoglycoside antibiotics were retrieved from the NCBI GenBank database https://www.ncbi.nlm.nih.gov/nuccore/?term= (accessed in 20 April 2024) via the accession codes provided in Table 1.
