# Supplementary material for: Combinatorial biosynthesis of novel aminoglycoside antibiotics via pathway engineering
Source: AMB Express. 2024 Sep 16;14:103. doi: 10.1186/s13568-024-01753-w (PMC11405602; doi:10.1186/s13568-024-01753-w)
Supplement: Supplementary file 1 — Supplementary Material 1 [file 13568_2024_1753_MOESM1_ESM.docx]

ketocyclitol AT (I)

ketocyclitol AT-II

G-6-P

1

3

2

4

5

6

OH

HO

HO

OH

O

2-deoxy-*scyllo*-inosose

2-deoxy-*scyllo*-3-inosamine

aminocyclitol 1-dehydrogenase

1

3

2

4

5

6

OH

HO

NH_2_

HO

NH_2_

1

3

2

4

5

6

OH

HO

HO

OH

NH_2_

1-keto-2,3-deoxy-3-amino-*scyllo*-inositol

1

3

2

4

5

6

OH

HO

HO

O

NH_2_

HO

HO

CH_2_O-P

O

OH

OH

UDP-D-glucosamine transferase

Hygromycin B

2-deoxy-*scyllo*-inosose synthase

1

3

2

4

5

6

OH

HO

NH_2_

NH_2_

HO

HO

CH_2_OH

O

NH_2_

O

3‘

6‘

2‘

4‘

1‘

UDP-D-glucosamine

Neomycins

Ribostamycins

Paromomycins

**NM group**

**KM group**

Gentamicins

Tobramycin

Kanamycins

Apramycin

Lividomycins

UDP-D-glucosamine synthase

+ TTP or UTP

glycosylations

glycosylations

**paromamine**

glycosylations

**(2-deoxy streptamine)**

**Figure S1**. The biosynthetic pathways for the synthesis of various ACAGAs. The intermediates, 2-deoxy streptamine and paromamine are indicated.
